# Effect of heat stress on the hypothalamic expression profile of water homeostasis‐associated genes in low‐ and high‐water efficient chicken lines

**DOI:** 10.14814/phy2.15972

**Published:** 2024-03-11

**Authors:** Loujain Aloui, Elizabeth S. Greene, Travis Tabler, Kentu Lassiter, Kevin Thompson, Walter G. Bottje, Sara Orlowski, Sami Dridi

**Affiliations:** ^1^ Center of Excellence for Poultry Science, Division of Agriculture University of Arkansas Fayetteville Arkansas USA; ^2^ Higher School of Agriculture of Mograne University of Carthage Zaghouan Tunisia; ^3^ Center for Agricultural Data Analyses, Divion of Agriculture University of Arkansas Fayetteville Arkansas USA

**Keywords:** broilers, gene expression, growth, hypothalamus, selection, water efficiency

## Abstract

With climate change, selection for water efficiency and heat resilience are vitally important. We undertook this study to determine the effect of chronic cyclic heat stress (HS) on the hypothalamic expression profile of water homeostasis‐associated markers in high (HWE)‐ and low (LWE)‐water efficient chicken lines. HS significantly elevated core body temperatures of both lines. However, the amplitude was higher by 0.5–1°C in HWE compared to their LWE counterparts. HWE line drank significantly less water than LWE during both thermoneutral (TN) and HS conditions, and HS increased water intake in both lines with pronounced magnitude in LWE birds. HWE had better feed conversion ratio (FCR), water conversion ratio (WCR), and water to feed intake ratio. At the molecular level, the overall hypothalamic expression of aquaporins (AQP8 and AQP12), arginine vasopressin (AVP) and its related receptor AVP2R, angiotensinogen (AGT), angiotensin II receptor type 1 (AT1), and calbindin 2 (CALB2) were significantly lower; however, CALB1 mRNA and AQP2 protein levels were higher in HWE compared to LWE line. Compared to TN conditions, HS exposure significantly increased mRNA abundances of AQPs (8, 12), AVPR1a, natriuretic peptide A (NPPA), angiotensin I‐converting enzyme (ACE), CALB1 and 2, and transient receptor potential cation channel subfamily V member 1 and 4 (TRPV1 and TRPV4) as well as the protein levels of AQP2, however it decreased that of AQP4 gene expression. A significant line by environment interaction was observed in several hypothalamic genes. Heat stress significantly upregulated AQP2 and SCT at mRNA levels and AQP1 and AQP3 at both mRNA and protein levels, but it downregulated that of AQP4 protein only in LWE birds. In HWE broilers, however, HS upregulated the hypothalamic expression of renin (REN) and AVPR1b genes and AQP5 proteins, but it downregulated that of AQP3 protein. The hypothalamic expression of AQP (5, 7, 10, and 11) genes was increased by HS in both chicken lines. In summary, this is the first report showing improvement of growth performances in HWE birds. The hypothalamic expression of several genes was affected in a line‐ and/or environment‐dependent manner, revealing potential molecular signatures for water efficiency and/or heat tolerance in chickens.

## Graphical abstract

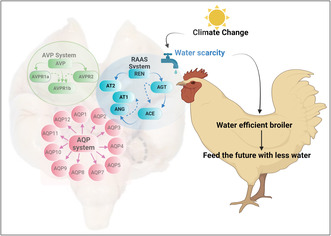

Divergently selected broilers for high water efficiency drunk less water during high environmental temperature and perform better than their low‐water efficient counterparts through modulation of hypothalamic AVP, RAAS, and aquaporin systems.

## 1 INTRODUCTION

Globally, poultry production provides high‐quality, nutritious, wholesome, and affordable animal proteins, and thereby supports the livelihoods and food security of billions of people worldwide (Lee, 2021; Mulder, 1997). Poultry has become and will remain the most consumed livestock commodity in the world. In fact, based on a 2011 report, the United Nations Food and Agriculture Organization (FAO) stated that world meat consumption will rise by 73% by 2050 (Alexandratos & Bruinsma, 2012), which will oblige poultry meat production to grow to 181 million metric tons or ~32% greater than 2022s value. While this surge is driven by the ever‐expanding world human population that is expected to reach between 9 and 10 billion by 2050 (Suzuki, 2019), the Global Harvest Initiative and the National Research Council (NRC) indicated that meeting this future high nutritional demand will be very challenging as the current global agricultural productivity (GAP) index falls 6% short of the target when compounded over 40 years (Goldstein et al., 2015; Smith et al., 2015). Furthermore, agriculture in general and poultry in particular are at the heart of planetary boundaries, global environmental risks (climate change and global warming) (Alley et al., 2005; Chen et al., 2011; Mora et al., 2013; Thompson et al., 2006), and limited natural resources (land, energy, and water) (Akpoti, Kabo‐Bah, & Zwart, 2019; Greve et al., 2018; McDonald et al., 2011; Schewe et al., 2014; Vorosmarty et al., 2000).

Built on unusual years of record‐breaking droughts and longer season temperatures, climate simulation predicts that global warming will only intensify and exponentially rise (Tollefson, 2022; Xu et al., 2018). The large, abrupt, and widespread heatwaves observed over the past decades, have adversely affected agricultural systems at all levels from crops, insects, to animal productions (Halupka et al., 2023; Jagermeyr et al., 2021; Moore et al., 2017; Radchuk et al., 2019; Sharma et al., 2022). Of particular interest, and due to their elevated core body temperature compared to mammals (~41°C vs. 37°C), high metabolic rate, feather covering, and lack of sweat glands, chickens (meat and egg types) are very sensitive to high environmental temperatures (Perini, Cendron, Rovelli, Castellini, & Cassandro, 2021). The detrimental effects of high environmental temperature on feed intake (Abdelli et al., 2021; Brugaletta et al., 2021; Tabler et al., 2020), growth (He et al., 2018; Liu et al., 2020; Wasti, Sah, & Mishra, 2020), welfare (Sesay, 2022; Shields, 2015), stress (Greene et al., 2022; Lin et al., 2006; Mahmoud & Edens, 2003), immune system (Jahanian & Rasouli, 2015; Liu et al., 2022; Molnar, Korosi, Balazs, & Gaspardy, 2021; Monson et al., 2018), and meat and egg quality (Dai et al., 2009; Dai et al., 2012; Leishman et al., 2021; Maynard et al., 2023; Zaboli et al., 2019) are well documented. Together, these effects consequently result in significant and heavy economic losses to the industry worldwide (Raspoet & Wang, 2022; St Pierre, Cabanov, & Schnitkey, 2003), in addition to the add capital investment such as fans, cooler pads, and water sprinklers.

The status quo pertaining to overcoming heat stress (HS) induced by high environmental temperatures across the world is the use of evaporative cooling system (pads, fogging, or low‐pressure misting systems), which readily use a significant amount of water. Moreover, a second form of water usage in bird live production is the water consumption that amounts yearly to millions of gallons (Pesti et al., 1985). According to the NRC (1994), water intake of chickens increases by about 7% for each 1°C rise above 21°C. Deeb and Cahaner (2002) on the other hand, reported that 10% higher water intake were recorded when birds were reared under HS (32°C) from Day 17 to 42 of age. Although it is arguable that the amount of water usage varies upon many factors (location, houses, density, management, bird strains, age, etc.), there is a consensus that water scarcity will be a burden and the greatest challenge in future unprecedented global water uncertainty and crisis. Indeed, global climate change occurring in tandem with land‐use changes causes profound alterations in the hydrological cycle and raises a series of water and feed availability and quality problems not only for animal agriculture but also for human consumption (Alcamo, Florke, & Marker, 2007). With this projected climate and population change, the global water‐scarce people will rise by 43%–50% to reach 6 billion by 2080 (Alcamo, Florke, & Marker, 2007). There is, therefore, a critical need for research focusing on overcoming these obstacles to sustainably feed the future by using less land and less water.

As for feed efficiency, water efficiency is a vital economic and agricultural trait, and can be defined as the efficacy of converting consumed water to weight. Water intake is tightly controlled at both central and peripheral levels through complex molecular mechanisms. At the hypothalamic levels, the thirst center, which is comprised of neurons that are endowed with an intrinsic ability to detect deficits in either intracellular or extracellular fluid volume, is localized mainly in the lamina terminalis (organum vasculosum of the lamina terminalis OVLT, subfornical organ SFO, and median preoptic nucleus MnOP). Several central pathways, including arginine‐vasopressin (AVP), aquaporins (AQPs), and renin‐angiotensin (RAAS) systems were extensively studied in mammals and have been shown to be involved in the regulation of water homeostasis [for review see (Dridi, 2022; McKinley & Johnson, 2004)]. However, avian regulation of water hemostasis is unexplored and the role of such pathways needs to be defined.

After four generations of divergent selection for water efficiency (Hiltz et al., 2021), we undertook the present study to determine the effect of chronic cyclic HS on feed and water intake, growth and hypothalamic expression of water homeostasis‐associated genes in low‐ (LWE) and high‐water efficient (HWE) broilers.

## 2 MATERIALS AND METHODS

### 2.1 Divergent selection for HWE or LWE broiler lines

The divergent genetic selection program for water efficiency was implemented in 2019. The base population utilized in the program was a 2015 Modern Random Bred (MRB) population (broiler (meat type) chickens, *Gallus domesticus*) (Hilts, 2021; Orlowski et al., 2020). Utilizing 24 sire families (sire family = 1 male and 3 females), the MRB families' water intake and growth were assessed for 4 weeks using a low‐flow water monitoring system (Hilts, 2021). Following the 4‐week measurement period, water conversion ratio (WCR) was calculated [WCR = water intake (g)/body weight gain (g)] for each sire family. The six families with the lowest WCR, indicating improved water efficiency, were dedicated to the HWE line, while the six families with the highest WCR were dedicated to the LWE line. As WCR is a direct measure, direct selection was implemented and selected families were grown to breeder age. For the F1 generation and every subsequent generation (F4), 0–4 weeks WCR was used as the single selection trait for sire family. To improve the trait, individual male WCR data was recorded from 4 to 6 weeks, following the initial sire family selection. HWE and LWE birds from Generation 4 were used in the present study.

### 2.2 Heat stress trial and experimental design

Eggs from the HWE and LWE lines were collected, incubated, and hatched at the University of Arkansas poultry hatchery. On the day of hatch, male chicks (240 chicks/line) were individually wing‐banded for line identification and weighed. The rational of using only males in the present study is to avoid gender effects and to reduce the number of main tested variables. There are already two main factors (line and environment) and their interaction, along with a random factor (chamber/pen). Females will be used in a future study. Chicks were allotted randomly by line and body weight‐matched group to 12 controlled environmental chambers in the Poultry Environmental Research Laboratory at the University of Arkansas (2 floor pens/chamber, 6 chambers/line, 20 birds/pen, density of 0.096 m^2^/bird). Each pen was covered with clean pine wood shavings and equipped with separate commercial hanging feeders and nipple water line attached to a low‐flow water monitoring system for each pen (Hilts, 2021). Water and three‐phase (starter, grower, and finisher) standard diets (3050 ME kcal/kg and 23.3% CP; 3125 ME kcal/kg and 21.1% CP; 3200 ME, kcal/kg and 19.1% CP, as fed basis, for starter, grower, and finisher, respectively) that are recommended by the industry were provided *at libitum*. The ambient temperature was gradually decreased from 32°C for Days 1–3, 31°C for Days 4–6, 29°C for Days 7–10, 27°C for Days 11–14, and 25°C thereafter. A relative humidity of ~30%–40% was maintained and the lighting program was 24 h light for the first 3 days, reduced to 23 h light:1 h dark from Day 4–7, and then reduced further to 18 h light:6 h dark thereafter. The environmental temperature and humidity were continuously recorded in each pen using HOBO pro V2 data loggers (ONSET, MA). At Day 29, birds were exposed to two environmental conditions: thermoneutral (25°C) or chronic cyclic heat stress (36°C for 9 h/day from 9:00 am to 6:00 pm) to mimic summer season in Arkansas, USA, which resulted in a total of four treatments in a 2 × 2 factorial split plot design (2 lines × 2 environmental conditions factorial designs, 6 pens/treatment, 120 birds/treatment). One day before the onset of HS, two chickens per pen were randomly selected and a Thermochron temperature logger (iButton, Embedded Data Systems, KY) was placed in the crop via oral gavage for continuous monitoring of core body temperature as previously described (Greene, Maynard, et al., 2021). Feed and water intake were measured at the pen level on a daily basis from D1 to 49 and corrected for mortality. Feed intake was determined by weighing the feeders before and after, and water intake was measured using automatic water monitoring systems (Hilts, 2021). At D 49, birds were euthanized by cervical dislocation and brains were collected from thermologger‐equipped chickens, and hypothalamuses (the main thirst center, McKinley & Johnson, 2004) were dissected as previously described (Piekarski et al., 2018).

### 2.3 Ethics statement

The present study was conducted in accordance with the recommendations in the guide for the care and use of laboratory animals of the National Institutes of Health and the protocol was approved by the University of Arkansas Animal Care and Use Committee (protocol # 23015).

### 2.4 Total RNA extraction, reverse transcription, and quantitative real‐time PCR

Total RNA extraction from the hypothalamuses was conducted as previously described (Dridi et al., 2012) with some modifications. In brief, hypothalamuses were harvested in 1.5 mL lysis buffer [10 mM Tris–HCl (pH 8.0), 140 mM NaCl, 1.5 mM MgCl_2_, 0.5% Igepal, 2 mM vanadyl ribonucleoside complex (VRC, ThermoFisher Scientific, Waltham, MA)] using Next Advance bullet blender gold bead homogenizer (Next Advance, Troy, NY). One‐tenth of the lysate was added to 1 mL Trizol reagent (ThermoFisher Scientific, Waltham, MA) for total RNA isolation according to manufacturer's recommendations. The rest of the lysate was used for immunoblotting as described in the following section. Total RNAs were treated with RQ1 DNAse and reverse transcribed (Quanta Biosciences, Gaithersburg, MD). RNA integrity and quality was evaluated by both OD260/OD280 nm absorption ratio (>1.8) and by using 1% agarose gel electrophoresis and RNA concentrations and purity were measured for each sample by Take 3 micro volume plate using Synergy HT multimode microplate reader (BioTek, Winooski, VT). For cDNA synthesis, total RNA (1 μg) was reverse transcribed using qScript cDNA SuperMix (Quanta Biosciences, Gaithersburg, MD) in a 20 μL total reaction. The reverse transcription reaction was performed at 42°C for 30 min followed by an incubation at 85°C for 5 min. Real‐time quantitative PCR (Applied Biosystems 7500 Real‐Time PCR system) was performed using 5 μL of 10X diluted cDNA, 0.5 μM of each forward and reverse specific primer, and PowerUp SYBR Green Master Mix (ThermoFisher Scientific, Rockford, IL) in a total 20 μL reaction as previously described (Cook et al., 2023; Dridi et al., 2012). Oligonucleotide primers specific for chicken aquaporins (AQP1, 2, 3, 4, 5, 7, 8, 9, 10, 11, and 12), arginine vasopressin (AVP) and its related receptors (AVPR1A, AVPR1B, and AVPR2), natriuretic peptide A (NPPA), renin‐angiotensin‐aldosterone (RAAS) system (renin [REN], angiotensinogen [AGT], angiotensin I‐converting enzyme [ACE], angiotensin II receptor type 1 and 2 [AT1 and AT2]), calbindin 1 and 2 (CALB1 and 2), secretin (SCT), and transient receptor potential cation channel subfamily V member 1 and 4 (TRPV1 and TRPV4), and ribosomal 18S as a housekeeping gene are compiled in Table 1. The cycling parameters for the qPCR amplification were as follows: an initial incubation at 50°C for 2 min, an initial denaturation step (95°C, 10 min) followed by 40 cycles of denaturation (95°C, 15 s) and annealing (58°C, 1 min). Melting curve analysis was applied, at the end of the amplification, by using the dissociation protocol (Sequence Detection system) to exclude contamination with unspecific PCR products. The PCR products were also confirmed by 2% agarose gel which exhibit only one definite band of the predicted size and by sequencing the amplified amplicons. There were no gel‐detected bands for the negative controls where the RT products were omitted. Relative expressions of target genes were determined by the 2^−ΔΔCt^ method (Schmittgen & Livak, 2008).

**TABLE 1:** Oligonucleotide QPCR primers.

Gene	Accession number^a^	Primer sequence (5′ → 3′)	Orientation	Product size (bp)
*AQP1*	NM_001039453	CAGCAACTCAGGACAACGTGAA	Forward	60
		CCATGGTCGCGATGGATAA	Reverse	
*AQP2*	NM_001292072	TTTGCAGCCTCCATGATGTG	Forward	56
		AGGACAGCCCGGGTGAA	Reverse	
*AQP3*	XM_424500	TGCTCCTGGTCCCTGACACT	Forward	58
		CTTTTGCCTTCCCATTGCA	Reverse	
*AQP4*	NM_001317827	CGCTCGCAGCAGCAGTAA	Forward	59
		ATGCTACCATGATGCTCTCACACT	Reverse	
*AQP5*	XM_001231780	TCCTGGCACACAACTGCAA	Forward	63
		AGAAAATGGCTCCGTTGACACT	Reverse	
*AQP7*	AB359225	CCCTGAAAGGCACACATGCT	Forward	58
		CCCATACCAATGCCCAGAAC	Reverse	
*AQP8*	XM_040684010	GCGCTGGGCAATGAGATC	Forward	56
		GTCATGCAGACCACGAGCAA	Reverse	
*AQP9*	NM_001293238	AACAGTGGCTGTGCCATGAA	Forward	65
		CCTGCAATGGCTGTGAAGAG	Reverse	
*AQP10*	XM_015298598	CACCATGGACTATGCGTCCTT	Forward	72
		TCTCGTGCCAGCTGGTTCT	Reverse	
*AQP11*	XM_040657583	GCGGTGTTGGCCAGAGTCT	Forward	61
		AATGGGATTCCGCCATCTC	Reverse	
*AQP12*	NM_001109679	CGTGTGCCTTTCGCTATGG	Forward	65
		GTGCCAAGCACCAGGAAGA	Reverse	
*AVP*	NM_205185	TCCGGGCACACTCAGCAT	Forward	81
		ATGTAGCAGGCGGAGGACAA	Reverse	
*AVPR1A*	NM_001110438	GCAGTATTGCAAACAGACACATGA	Forward	65
		CCCACAATGCGAGTGGTTCT	Reverse	
*AVPR1B*	NM_001031498	TCTCCCGCGCCAAGATC	Forward	58
		ACGTAGGCCACCACGATGA	Reverse	
*AVPR2*	NM_001031479	GTTCGCACTAGCAGGAGGAGAT	Forward	75
		GCCAGCCAGCAATAAACCA	Reverse	
*NPPA*	NM_204925	TGACCTGCAAGAGCCTCAAA	Forward	61
		ATCGCTGTCATCTGTGAGTTCTG	Reverse	
*REN*	XM_040691529	TGCCGGGTCTTTCCATCA	Forward	65
		GGCATTTTCCACCACCAGTAG	Reverse	
*AGT*	NM_001396391	CAGGGTTTGCTGGGATTTGT	Forward	55
		CCCTGGAGGTGCAATTGG	Reverse	
*ACE*	NM_001167732	ACCCAAAGCAGAAAAGAGCTATTTAT	Forward	69
		GCCGGTGCCTGAATTTCTC	Reverse	
*AT1*	NM_205157	GCCTTAGCATCGACCGCTAT	Forward	62
		GGTACGTCGGATTCGTGACTTC	Reverse	
*AT2*	XM_040670971	GGAAACCCTCCAGATCCTCTATACA	Forward	62
		GCGGCGAGCGTAACACA	Reverse	
*CALB1*	NM_205513	TCTTTCTTCTTCCCGTCTTCCTT	Forward	67
		TGGAACAAAGATGGTGCAAAGT	Reverse	
*CALB2*	X62866	AGGCCAAGCTGCAGGAGTAC	Forward	58
		TCCCCGTTCATGTCAAACATC	Reverse	
*SCT*	NM_001024833	GAGGCACTCGGATGGACTGT	Forward	61
		CACCTGAGCGTTTCCTCTCAT	Reverse	
*TRPV1*	NM_204572	GCACTCCACATTGCCATTGA	Forward	66
		TGCTCCATTCTGGACCAAGAG	Reverse	
*TRPV4*	NM_204692	CAAAGACCTGTTCCGCTTCCT	Forward	61
		TGCTGAGGCGTAGCCAATC	Reverse	
*18S*	AF173612	TCCCCTCCCGTTACTTGGAT	Forward	60
		GCGCTCGTCGGCATGTA	Reverse	

Abbreviations: *ACE*, angiotensin I‐converting enzyme; *AGT*, angiotensinogen; *AQP*, aquaporin; *AT*, Angiotensin II receptor; *AVP*, arginine vasopressin; *AVPR*, AVP receptor; *CALB*, calbindin; *NPPA*, natriuretic peptide A; *TRPV*, transient receptor potential cation channel subfamily V.

^a^ Accession number refer to GenBank (NCBI) and primers were synthesized by Integrated DNA Technologies (IDT, Coralville, IA, USA).

### 2.5 Western blot

Total proteins were quantified and subjected to western blot analysis as we previously described (Dridi et al., 2012). Briefly, after homogenization, hypothalamic proteins were quantified using a Bradford assay kit (Bio‐Rad, Hercules, CA), BSA as standard, and a Synergy HT multimode microplate reader (Biotek Agilent, Winooski, VT). Proteins (70–100 μg) were run in gradient Bis‐Tris gels (4%–12%, Life Technologies, Carlsbad, CA), transferred to polyvinylidene difluoride (PVDF) membranes, and blocked with tris‐buffered saline, nonfat milk (5%), and Tween 20 (TBST) for 1 h at room temperature. After washed with TBST, the membranes were incubated with primary antibodies (dilution at 1:500 or 1:1000) overnight at 4°C. Primary antibodies used were rabbit anti‐AQPs (AQP1 # A15030, AQP2 # A16209, AQP3 # A2838, AQP4 # A2887, AQP10 # A2888, ABclona, Woburn, MA), rabbit anti‐AQP5 (#orb665771, Biorbyt, Cambridge, UK), and rabbit anti‐glyceraldehyde 3‐phosphate dehydrogenase (GAPDH, #NB300‐327, Novus Biologicals, Centennial, CO) as a housekeeping protein. Pre‐stained molecular weight marker (Precision Plus Protein Dual Color, BioRad, Hercules, CA) was used as a standard and as indicator for transfer efficiency. The secondary anti‐rabbit IgG‐HRP‐linked antibody (#7074S, Cell Signaling, Technology, Danvers, MA) was used at 1:5000 dilution for 1 h at room temperature. The signal was visualized by enhanced chemiluminescence (Super ECL, ABP Biosciences, Beltsville, MD) and captured by FluorChem M MultiFluor System (Proteinsimple, Santa Clara, CA). Image Acquisition and Analysis were performed by AlphaView software (Version 3.4.0, 1993–2011, Proteinsimple, Santa Clara, CA).

### 2.6 Statistics

Phenotypic and performance data (*n* = 120/line/environment) were analyzed as split plot using the mixed model personality of the Fit Model platform in JMP Pro v 17.1 (SAS Institute, Cary, NC). The model included the fixed factors (line, environment, and their interaction) and the random effect of pen nested in chamber. The means were compared by Tukey's HSD multiple comparison test. Growth performance data (FI, WC, BW, FCR, WCR, etc.) were corrected for mortality and bird was used as the experimental unit. Hypothalamic protein and gene expression data (n = 4–6 and 12/line/environment, respectively) were analyzed by two‐way ANOVA. If ANOVA revealed significant effects, the means were compared by Tukey's HSD multiple comparison test using Graph Pad Prism version 9.00 for Windows (Graph Pad Software, La Jolla California, USA). Data are presented as the mean ± standard deviation and the statistical significance was set at *p* < 0.05.

## 3 RESULTS

### 3.1 Divergent responses of HWE and LWE lines to thermal stress: Core body temperature, growth and water intake

**FIGURE 1:**
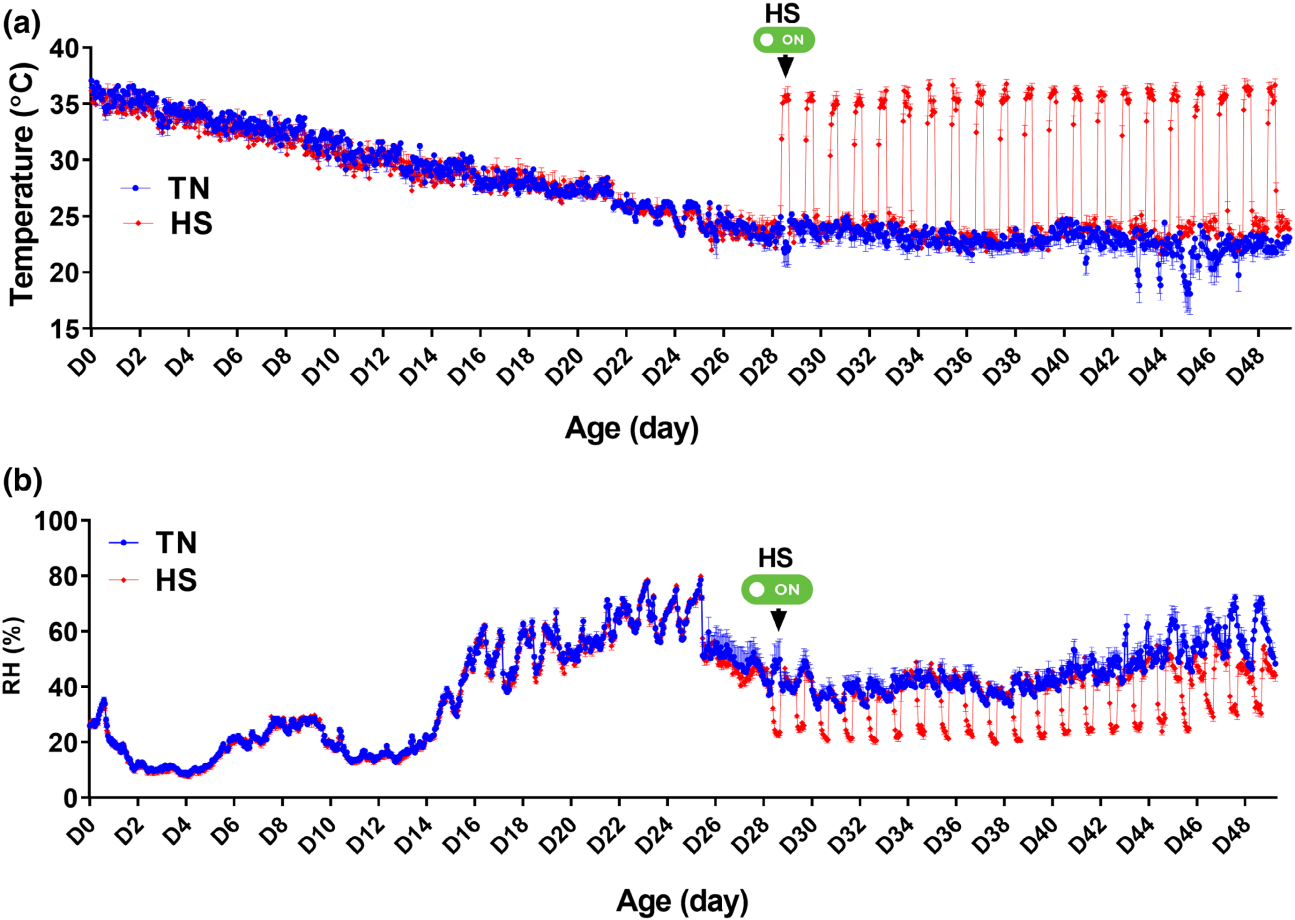
Temperature (a) and relative humidity (RH, b) fluctuations in the environmental chambers during the cyclic heat stress experiment. HWE and LWE birds were raised by line under recommended conditions from Day 0 to 28. From Day 29 to 42, birds either raised at thermoneutral temperature (25°C) or exposed to chronic cyclic heat stress (35°C for 9 h/day from 9:00 am to 6:00 pm). Data are presented as mean ± SD (*n* = 6 pens/group).

**FIGURE 2:**
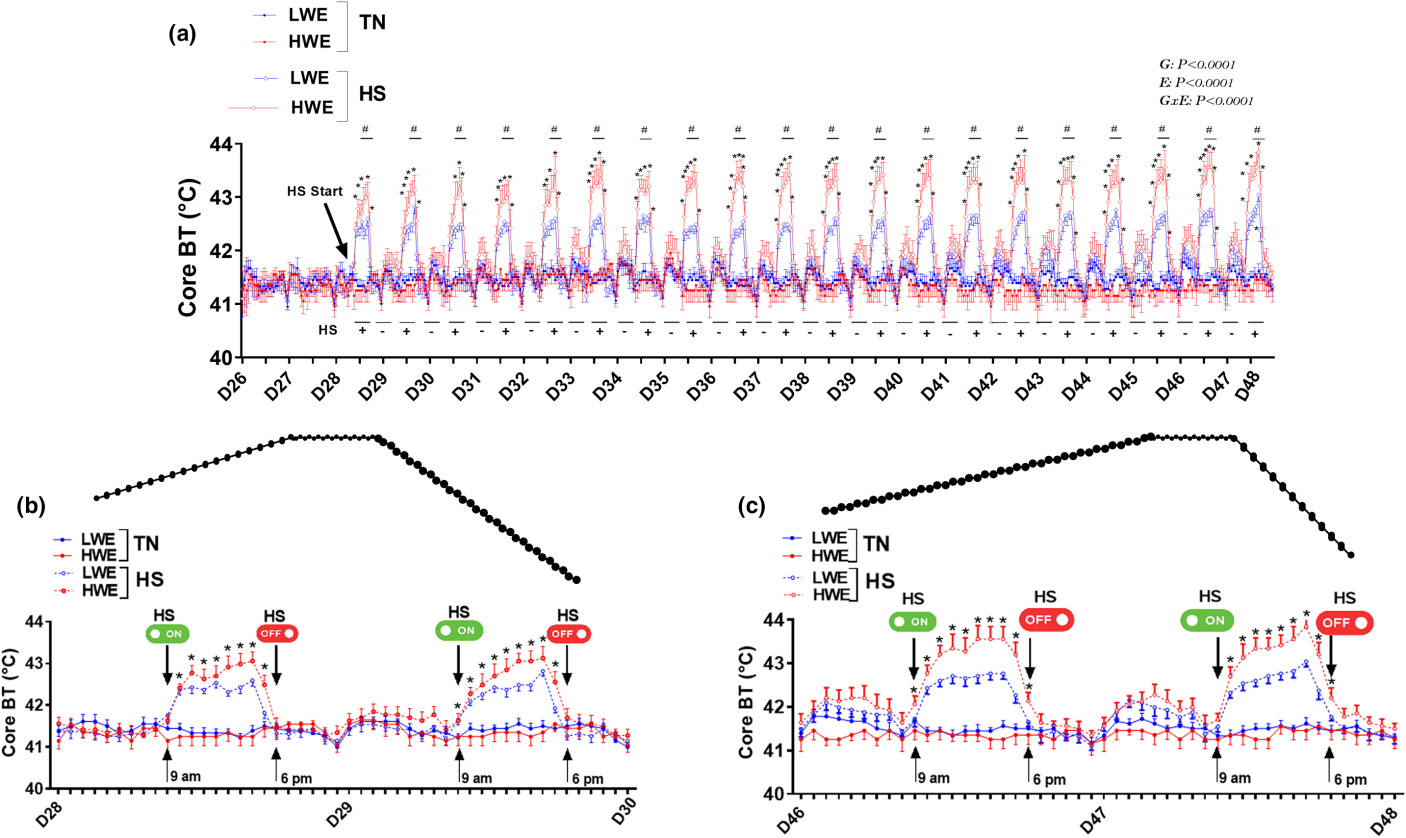
Core body temperature fluctuations during chronic cyclic heat stress (HS) experiment. (a) represents the whole HS period from Day 29 to 41, (b, c) represent a detailed daily variation from Day 28 to30 and Day 46 to 48, respectively. Birds were exposed to two environmental conditions: TN, 25°C or HS (35°C, 9 h/d) in a 2 × 2 factorial design as described in materials and methods. BT, body temperature; HS, heat stress condition; HWE, high‐water efficient chickens; LWE, low‐water efficient chickens; TN, thermoneutral condition. #Indicates significant difference between TN and HS conditions and * indicates difference between HWE and LWE during HS conditions.

The experiment was conducted in the controlled environmental chambers in the Poultry Environmental Research Laboratory at the University of Arkansas from April to June 2023 for 49 days. As depicted in Figure 1a, the cyclic temperature inside the environmental chambers was successfully maintained as planned (36°C for 9 h/day from 9:00 am to 6:00 pm, and 25°C during the rest of the day). Although the temperature in the TN chambers were maintained as planned, there was larger variabilities across chambers at the end of the trial due to high external environmental temperatures that force the automated air‐conditioning to cycle more frequently with some time variations. The RH inside the chambers averaged between 15% and 40% from Day 1 to 29, and then cyclically between 20% during HS period and ~40% during the rest of the day from Day 29 and forward (Figure 1b). The cyclic 11°C increase in ambient temperature significantly elevated core body temperatures of both chicken lines by ~ 0.5–2°C (Figure 2a). However, as shown in the daily plots, the amplitude of the core body temperature was significantly higher by ~ 0.5°C at the beginning of HS period and reached ~ 1°C at the end of HS in HWE compared to their LWE counterparts (Figure 2b,c). This elevation occurred within 2 h of the daily rise in environmental temperature and returned to TN levels as the ambient temperature returned to 25°C (Figure 2a–c).

**FIGURE 3:**
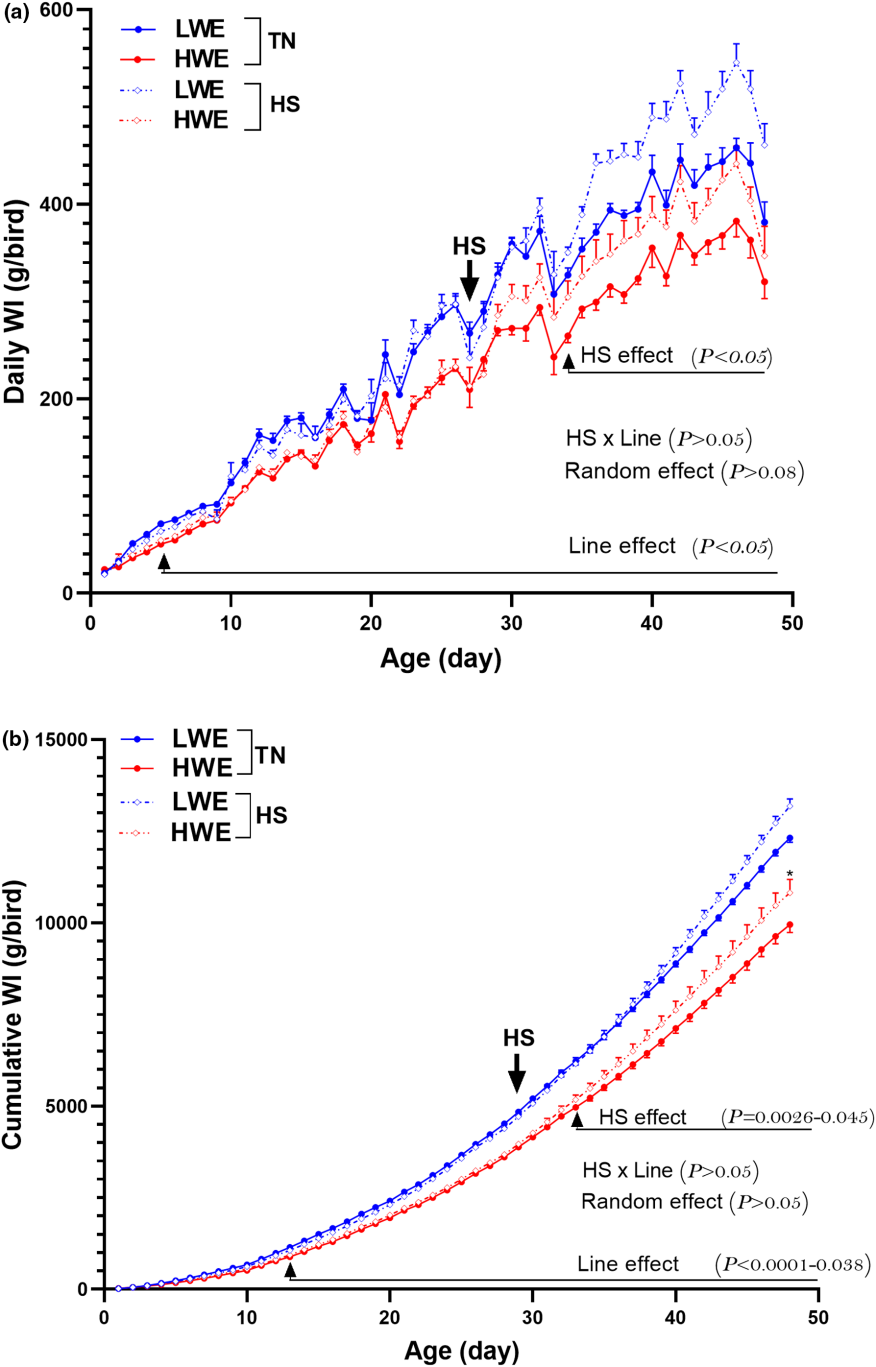
Water intake of HWE and LWE chickens maintained at TN conditions or exposed to chronic cyclic HS. (a) daily WI and (b) cumulative WI. HS, heat stress condition; HWE, high‐water efficient; LWE, low‐water efficient; TN, thermoneutral condition; WI, water intake. The arrow indicates the start of the HS period.

As shown in Figure 3 and Tables 3 and 4, HWE line drank significantly less water than their LWE counterparts during both TN and HS conditions. HS increased water intake in both lines, but the effect was more pronounced in the LWE birds (1000.24 vs. 796.58 g/bird, Tables 3 and 4). HS significantly increased WCR in both lines, however the amplitude was higher in LWE compared to HWE birds (56 vs. 63 points for the whole period) (Table 4).

**FIGURE 4:**
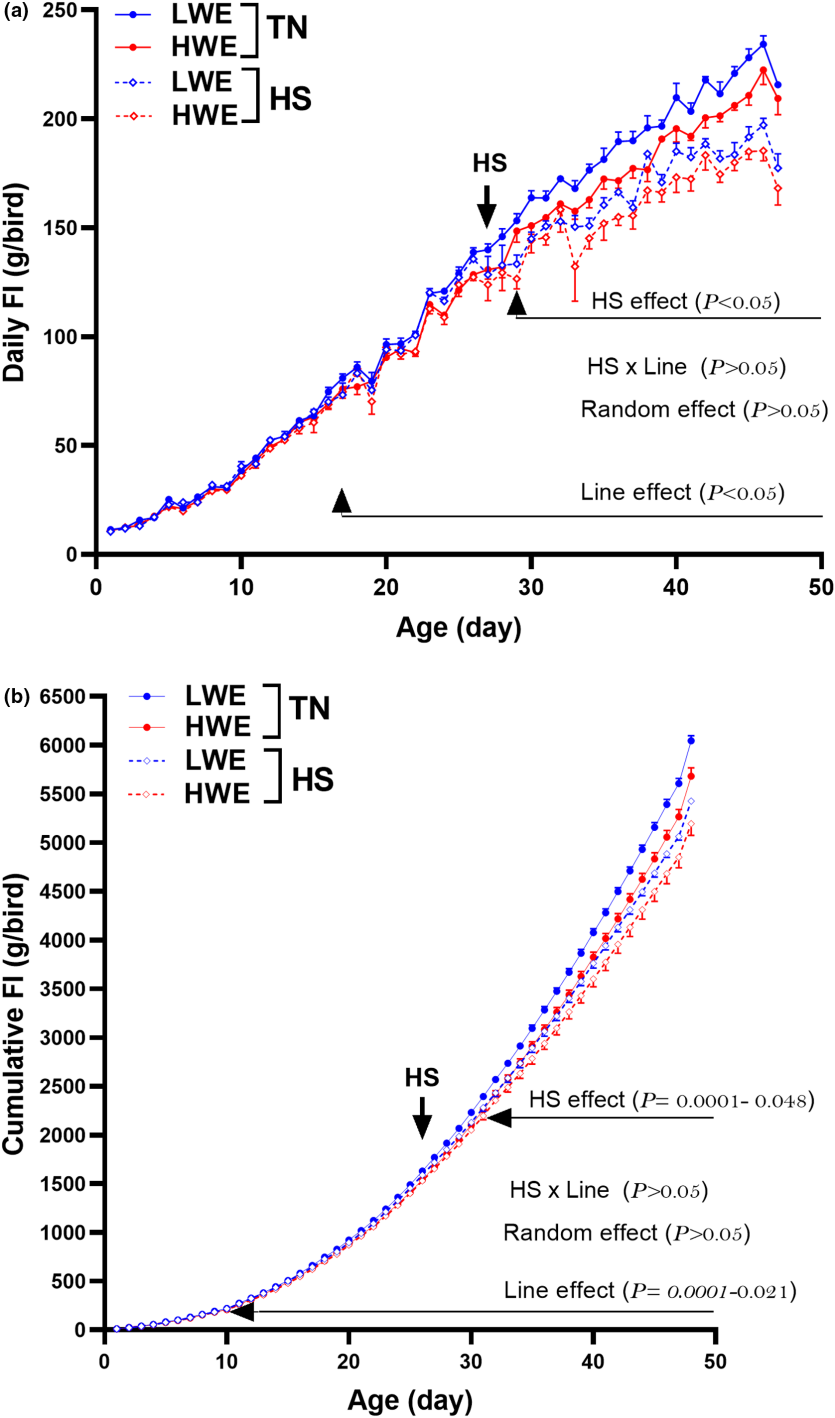
Feed intake of HWE and LWE chickens maintained at TN conditions or exposed to chronic cyclic HS. (a) daily FI and (b) cumulative FI. FI, feed intake; HS, heat stress condition; HWE, high‐water efficient; LWE, low‐water efficient; TN, thermoneutral condition. The arrow indicates the start of the HS period.

During the 4‐week‐pre‐HS period, there was a line effect only, but neither stress nor interaction effects, on both water and feed intake. The significant effect of line was discerned on Day 10 and Day 4 for FI (*P* = 0.0001–0.048) and WI (*P* = 0.0001–0.038), respectively (Figures 3 and 4). HS exposure (4–7 weeks), as expected, significantly depressed feed intake in both lines, however the effect was less pronounced in LWE compared to their HWE counterparts (433.87 g vs. 508.16 g, Figure 4a,b, Table 4), yet FI of HS‐HWE line was significantly lower than that of HS‐LWE birds and this trend remains for the whole HS exposure period (4–7 weeks, Table 2). This, in turn, resulted in a significant line effect on the whole period FCR with 6‐ and 9‐points better FCR for HWE compared to LWE line under TN and HS conditions, respectively (Table 4). There was no line effect, but a significant HS effect on the whole period BWG with a more pronounced reduction in LWE compared to HWE birds (283.66 vs. 277.18 g, Table 4). The water to feed (W/F) ratio was significantly lower in HWE compared to LWE chickens under TN condition (Tables 2, 3, 4). Under HS exposure, the W/F ratio significantly increased in both lines with almost the same amplitude (Table 3).

**TABLE 2:** Growth performance of HWE and LWE chicken lines during the preheat stress (0–4 weeks) period.

Lines	HWE		LWE		*p* value		
Environment\Parameters	TN	HS	TN	HS	E	G	E × G
BWG (g/bird)	1210.29 ± 16.53	1209.91 ± 17.14	1255.28 ± 16.83	1224.33 ± 20.06	0.39	0.10	0.4
FI (g/bird)	1804.13 ± 25.18	1780.99 ± 28.82	1914.77 ± 23.65	1850.95 ± 29.94	0.10	0.009	0.45
WI (g/bird)	3184.94 ± 40.33	3216.05 ± 53.92	3900.85 ± 86.66	3827.81 ± 111.75	0.78	<0.0001	0.50
FCR	1.49 ± 0.01	1.47 ± 0.01	1.52 ± 0.01	1.51 ± 0.008	0.11	0.003	0.60
WCR	2.63 ± 0.02	2.65 ± 0.03	3.10 ± 0.07	3.12 ± 0.08	0.72	<0.0001	>0.99
W/F ratio	1.76 ± 0.02	1.80 ± 0.02	2.03 ± 0.04	2.06 ± 0.05	0.25	<0.0001	0.86

	Main effect (G)^a^		*p* value
	HWE	LWE	
FI (g/bird)	1810.42 ± 21.9	1882.86 ± 20.6*	0.02
WI (g/bird)	3200.50 ± 32.4	3864.33 ± 68.3*	<0.0001
FCR	1.48 ± 0.01	1.51 ± 0.006*	0.01
WCR	2.64 ± 0.01	3.11 ± 0.05*	<0.0001
W/F ratio	1.76 ± 0.02	2.05 ± 0.03*	<0.0001

Abbreviations: E, environment; E × G, interaction; FCR, feed conversion ratio; FI, feed intake; G, genotype (line); HS, heat stress; HWE, high‐water efficient; LWE, low‐water efficient; TN, thermoneutral; WCR, water conversion ratio; W/F, water to feed ratio; WI, water intake.

^a^ When the interaction (G × E) is not significant, the main effects (G and/or E) were analyzed separately using Student's *t*‐test. *indicates significant differences at *p* < 0.05.

**TABLE 3:** Growth performance of HWE and LWE chicken lines during the heat stress (4–7 weeks) period.

Lines	HWE		LWE		*p* value		
Environment\Parameters	TN	HS	TN	HS	E	G	E × G
BWG (g/bird)	2018.57 ± 51.83	1741.77 ± 57.22	2054.01 ± 31.80	1801.30 ± 26.13	<0.0001	0.29	0.78
FI (g/bird)	3669.68 ± 60.89	3161.52 ± 121.26	3914.97 ± 36.98	3481.10 ± 78.10	<0.0001	0.002	0.64
WI (g/bird)	6343.46 ± 174.34	7140.04 ± 314.70	7800.97 ± 107.83	8801.21 ± 158.82	0.0003	<0.0001	0.62
FCR	1.82 ± 0.02	1.82 ± 0.04	1.91 ± 0.03	1.94 ± 0.05	0.68	0.009	0.68
WCR	3.14 ± 0.06	4.09 ± 0.07	3.81 ± 0.10	4.89 ± 0.06	<0.0001	<0.0001	0.39
W/F ratio	1.73 ± 0.02	2.26 ± 0.08	1.99 ± 0.03	2.53 ± 0.06	<0.0001	<0.0001	0.92

	Main effect (E)^a^		*p* value
	TN	HS	
BWG (g/bird)	2036.29 ± 41.80	1771.53 ± 41.67*	<0.0001
FI (g/bird)	3792.32 ± 48.93	3321.31 ± 99.68*	<0.0001
WI (g/bird)	7072.21 ± 141.08	7970.62 ± 236.76*	0.0012
WCR	3.47 ± 0.08	4.49 ± 0.06*	<0.0001
W/F ratio	1.86 ± 0.02	2.39 ± 0.04*	<0.0001

	Main effect (G)^a^		*p* value
	HWE	LWE	
FI (g/bird)	3415.60 ± 91.07	3698.03 ± 57.54*	0.009
WI (g/bird)	6741.75 ± 244.52	8301.09 ± 133.32*	<0.0001
FCR	1.82 ± 0.03	1.92 ± 0.04*	0.046
WCR	3.61 ± 0.06	4.35 ± 0.08*	<0.0001
W/F ratio	1.99 ± 0.05	2.26 ± 0.04*	<0.0001

Abbreviations: E, environment; E × G, interaction; FCR, feed conversion ratio; FI, feed intake; G, genotype (line); HS, heat stress; HWE, high‐water efficient; LWE, low‐water efficient; TN, thermoneutral; WCR, water conversion ratio; W/F, water to feed ratio; WI, water intake.

^a^ When the interaction (G × E) is not significant, the main effects (G and/or E) were analyzed separately using Student's *t*‐test. *indicates significant differences at *p* < 0.05.

**TABLE 4:** Growth performance of HWE and LWE chicken lines during the whole rearing (0–7 weeks) period.

Lines	HWE		LWE		*p* value		
Environment\Parameters	TN	HS	TN	HS	E	G	E × G
BWG (g/bird)	3228.87 ± 58.59	2951.69 ± 65.36	3309.30 ± 37.49	3025.64 ± 10.06	<0.0001	0.12	0.94
FI (g/bird)	5473.82 ± 76.54	4942.52 ± 141.44	5829.75 ± 53.20	5332.05 ± 81.91	<0.0001	0.0008	0.86
WI (g/bird)	9528.40 ± 213.95	10356.10 ± 356.37	11701.82 ± 107.86	12629.02 ± 186.56	0.001	<0.0001	0.83
FCR	1.70 ± 0.01	1.67 ± 0.02	1.76 ± 0.01	1.76 ± 0.03	0.44	0.0009	0.44
WCR	2.95 ± 0.03	3.51 ± 0.06	3.54 ± 0.06	4.17 ± 0.06	<0.0001	<0.0001	0.52
W/F ratio	1.74 ± 0.02	2.10 ± 0.05	2.01 ± 0.02	2.37 ± 0.04	<0.0001	<0.0001	0.99

	Main effect (E)^a^		*p* value
	TN	HS	
BWG (g/bird)	3269.08 ± 48.04	2988.66 ± 37.71*	<0.0001
FI (g/bird)	5651.78 ± 64.87	5137.28 ± 111.67*	<0.0001
WI (g/bird)	10615.11 ± 160.90	11492.56 ± 271.46*	0.005
WCR	3.24 ± 0.04	3.84 ± 0.06*	<0.0001
W/F ratio	1.87 ± 0.02	2.23 ± 0.04*	<0.0001

	Main effect (G)^a^		*p* value
	HWE	LWE	
FI (g/bird)	5208.17 ± 108.99	5580.9 ± 67.55*	0.003
WI (g/bird)	9942.25 ± 285.16	12165.42 ± 147.21*	<0.0001
FCR	1.68 ± 0.01	1.76 ± 0.02*	0.0004
WCR	3.23 ± 0.04	4.73 ± 0.06*	<0.0001
W/F ratio	1.92 ± 0.03	2.19 ± 0.03*	<0.0001

Abbreviations: E, environment; E × G, interaction; FCR, feed conversion ratio; FI, feed intake; G, genotype (line); HS, heat stress; HWE, high‐water efficient; LWE, low‐water efficient; TN, thermoneutral; WCR, water conversion ratio; W/F, water to feed ratio; WI, water intake.

^a^ When the interaction (G × E) is not significant, the main effects (G and/or E) were analyzed separately using Student's *t*‐test. * indicates significant differences at *p* < 0.05.

### 3.2 Effects of HS on the hypothalamic expression of AQP families in HWE and LWE lines

**FIGURE 5:**
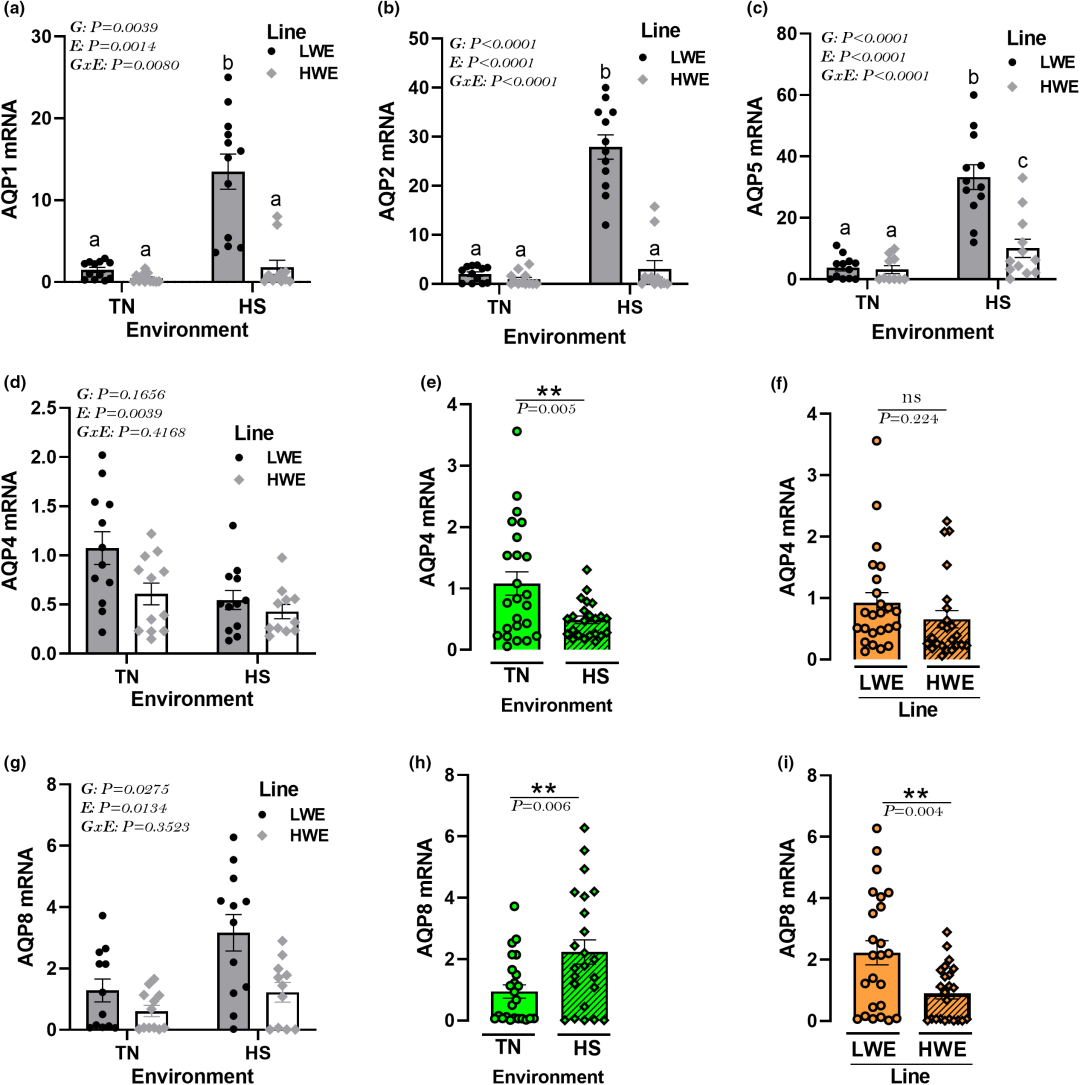
Effects of HS exposure on hypothalamic expression of orthodox aquaporin genes in HWE and LWE chickens. Relative expression of AQP1 (a), AQP2 (b), AQP5 (c), AQP4 (d–f), and AQP8 (g–i) gene was determined by qPCR and analyzed by 22^−ΔΔCt^ method (Schmittgen & Livak, 2008) using LWE‐TN group as a calibrator. Data are presented as mean ± SD (*n* = 12 birds/group) and analyzed by two‐way ANOVA and Tukey's HSD multiple comparison test. If the G by E interaction is not significant, the main effect (G or E) was analyzed separately by Student's *t*‐test. Asterisk (*) and different letters indicate significant difference at *p* < 0.05. AQP, aquaporin; E, environment; G, genotype or line; G × E, genotype by environment interaction; HS, heat stress; HWE, high‐water efficient; LWE, low‐water efficient; TN, thermoneutral.

**FIGURE 6:**
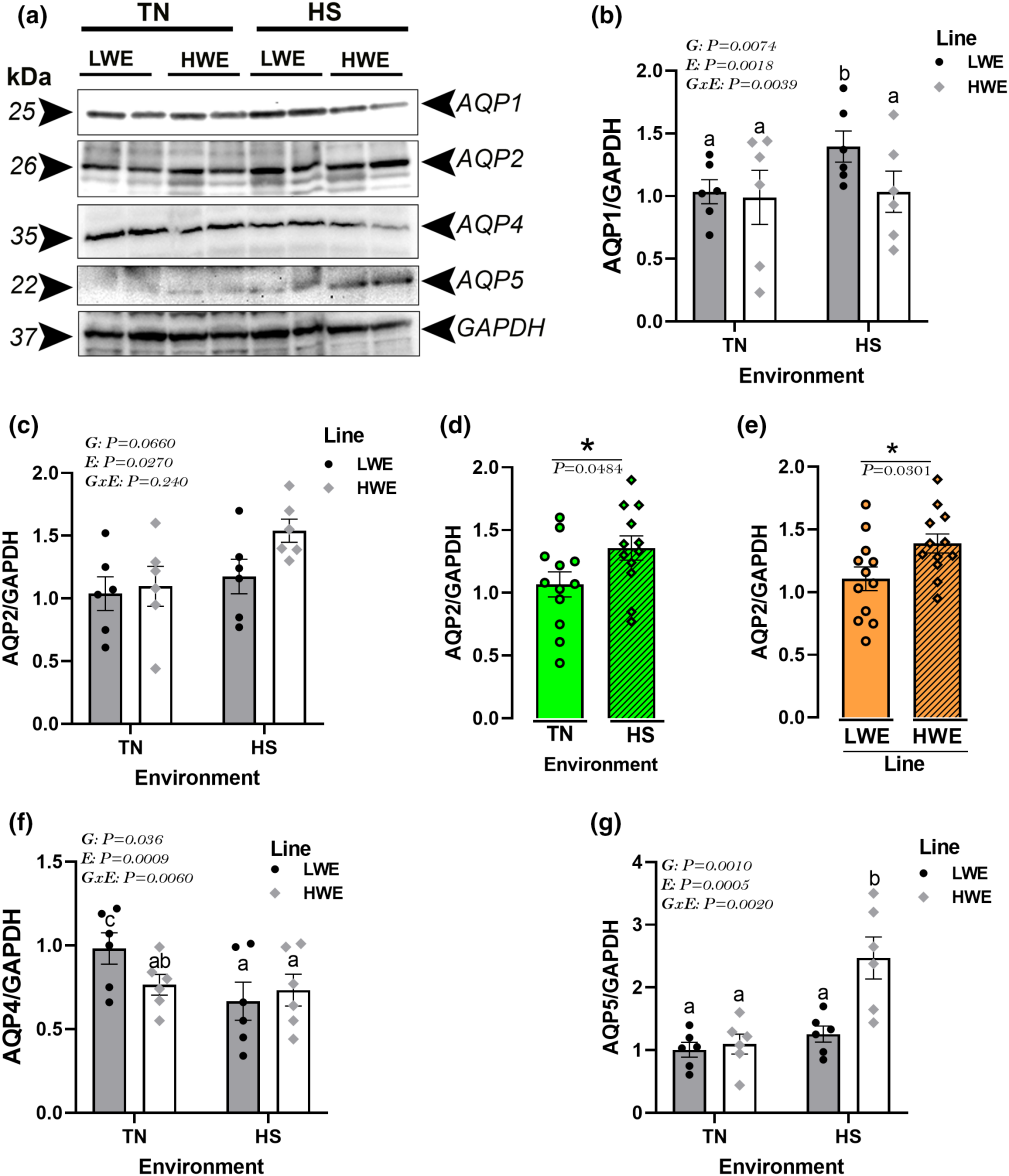
Effects of HS exposure on hypothalamic expression of orthodox aquaporin proteins in HWE and LWE chickens. Protein expression was determined by immunoblot as described in materials and methods. The signal was visualized by ECL plus, captured by FluorChem M MultiFluor System, and the image analysis was performed by AlphaView software. Data are presented as mean ± SD (*n* = 4–6 birds/group) and analyzed by two‐way ANOVA and Tukey's HSD multiple comparison test. If the G by E interaction is not significant, the main effect (G or E) was analyzed separately by Student's *t*‐test. Asterisk (*) and different letters indicate significant difference at *p* < 0.05. AQP, aquaporin; E, environment; G, genotype or line; G × E, genotype by environment interaction; HS, heat stress; HWE, high‐water efficient; LWE, low‐water efficient; TN, thermoneutral.

HS exposure significantly increased mRNA abundances of hypothalamic orthodox AQPs (AQP1, 2, and 5) in LWE but not in HWE birds (Figure 5a–c). There was no significant effect of the environment by line (G × E) interaction for AQP4 and AQP8 (Figure 5d,g), and the main effects were analyzed separately. As shown in Figures 5e,h, HS significantly downregulated hypothalamic AQP4, but upregulated that of AQP8 gene expression compared to TN conditions. The hypothalamic expression of AQP8, but not AQP4, was significantly downregulated in HWE compared to LWE chickens (Figure 5f,i). The immunoblot analyses showed that HS upregulated the hypothalamic protein levels of AQP1 and downregulated that of AQP4 in LWE birds (Figure 6a–c); however, it increased AQP5 protein expression in HWE birds compared to TN conditions (Figure 6a,d). There was no interaction effect on hypothalamic AQP2 protein expression, and it was upregulated in both HS condition (*P* = 0.0484) and HWE birds (*P* = 0.0301) compared to TN and LWE line, respectively (Figure 6c–e).

**FIGURE 7:**
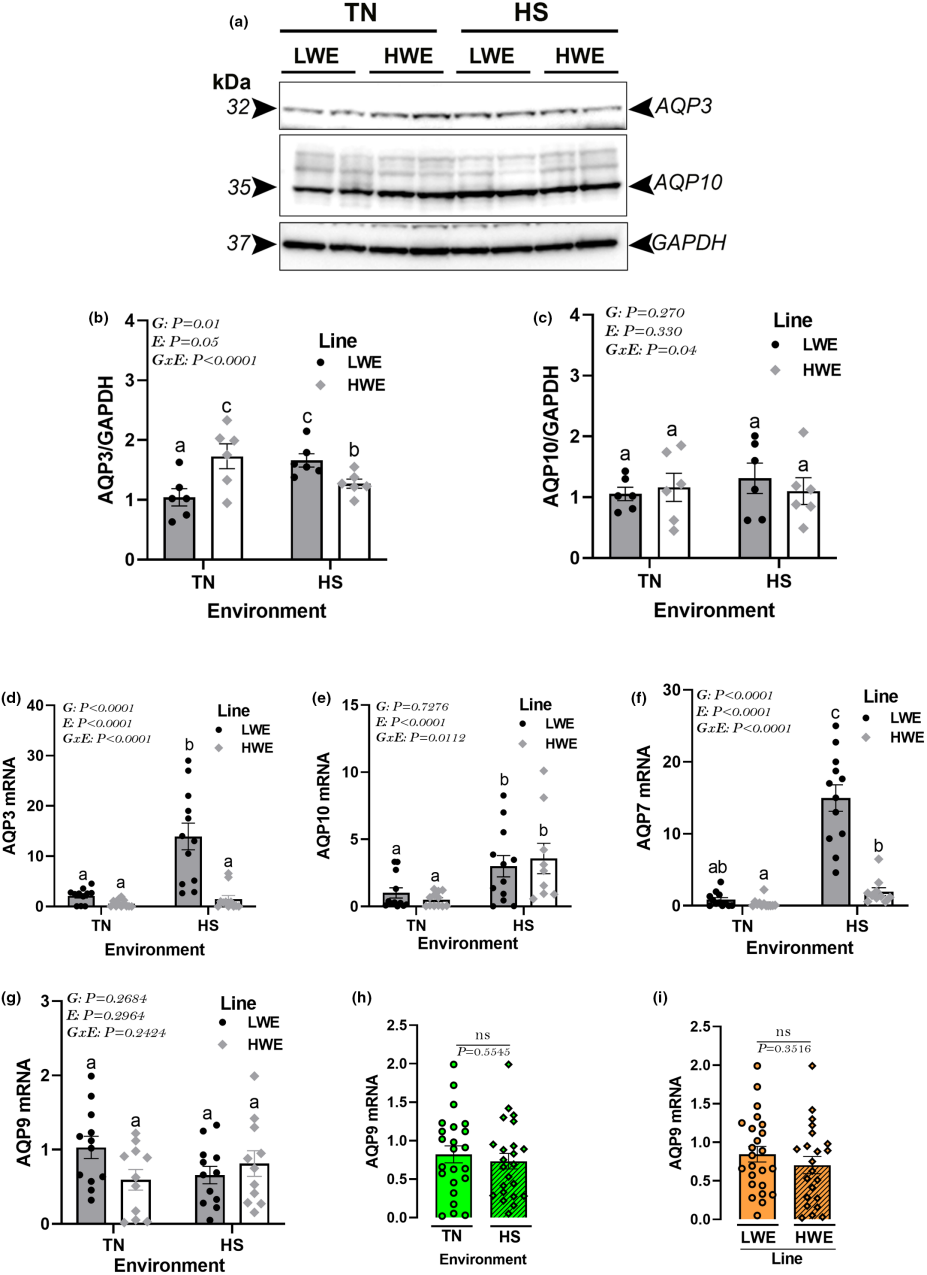
Effects of HS exposure on hypothalamic expression of aquaglyceroporins in HWE and LWE chickens. Protein levels of AQP3 and AQP10 were measured by western blot (a–c). mRNA abundances of AQP3 (d), AQP10 (e), AQP7 (f), and AQP9 (g–i) were determined by qPCR. Data are presented as mean ± SD (*n* = 4–6 and 12 birds/group for western blot and qPCR, respectively) and analyzed by two‐way ANOVA and Tukey's HSD multiple comparison test. Different letters indicate significant difference at *P* < 0.05. AQP, aquaporin; E, environment; G, genotype or line; G × E, genotype by environment interaction; HS, heat stress; HWE, high‐water efficient; LWE, low‐water efficient; TN, thermoneutral.

The hypothalamic expression of the aquaglyceroporin AQP3 protein was significantly increased in LWE, but decreased in HWE birds by HS exposure, which resulted in a significant G × E interaction (Figure 7a,b). AQP10 protein levels remained unchanged between all studied groups (Figure 7a,c). At gene expression levels, HS upregulated the hypothalamic expression of AQP3 in LWE, and AQP10 and AQP7 in both lines (Figure 7d–f). mRNA abundances of AQP9 did not differ between all studied groups (Figure 7g–i).

**FIGURE 8:**
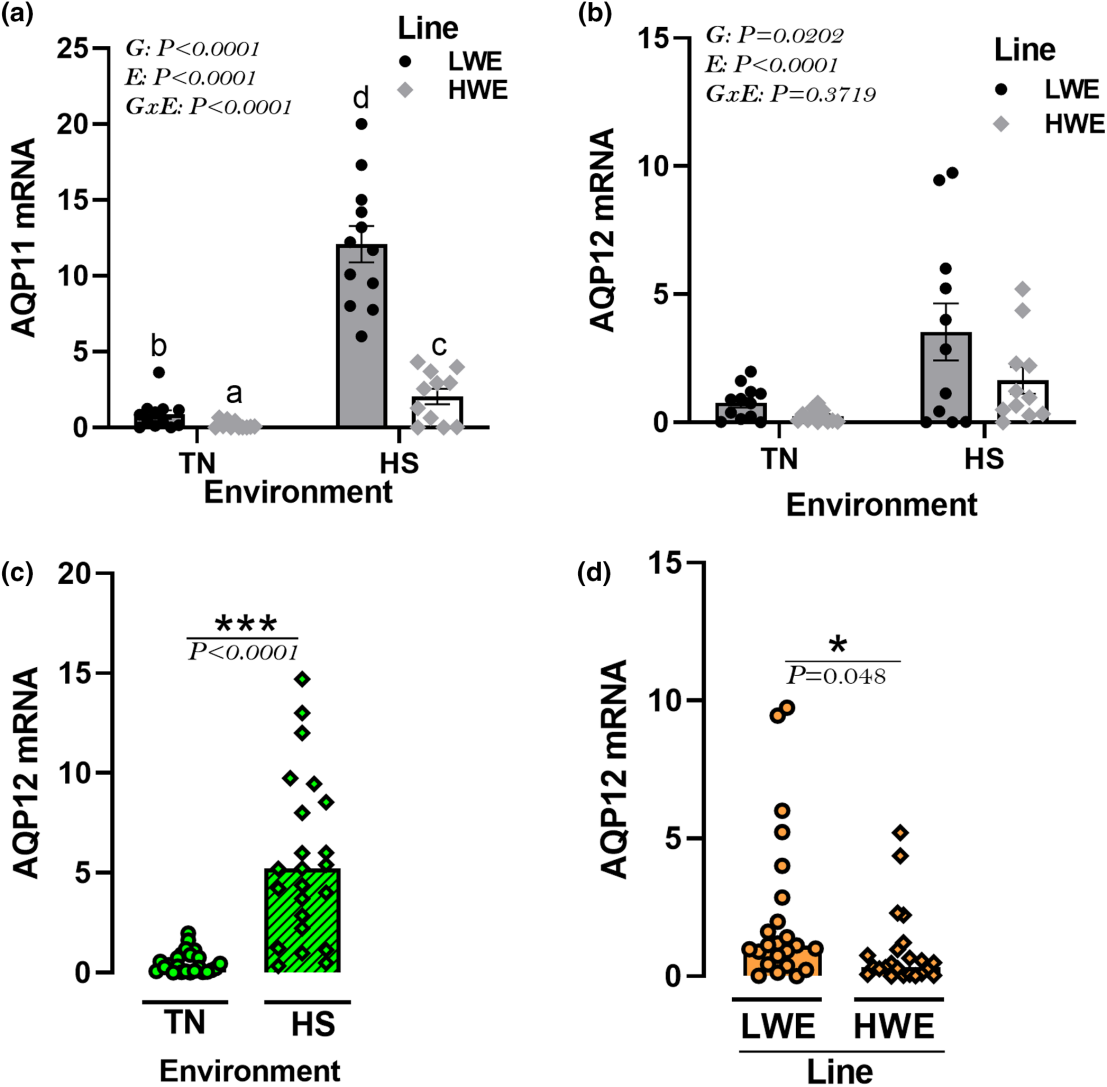
Effects of HS exposure on hypothalamic expression of unorthodox aquaporin genes in HWE and LWE chickens. Relative expression of AQP11 (a) and AQP12 (b‐d) gene was determined by qPCR and analyzed by 2^−ΔΔCt^ method (Schmittgen & Livak, 2008) using LWE‐TN group as a calibrator. Data are presented as mean ± SD (*n* = 12 birds/group) and analyzed by two‐way ANOVA and Tukey's HSD multiple comparison test. If the G by E interaction is not significant, the main effect (G or E) was analyzed separately by Student's *t*‐test. Asterisk (*) and different letters indicate significant difference at *p* < 0.05. AQP, aquaporin; E, environment; G, genotype or line; GxE, genotype by environment interaction; HS, heat stress; HWE, high‐water efficient; LWE, low‐water efficient; TN, thermoneutral.

The hypothalamic expression of superaquaporin AQP11 was affected by HS with a significant upregulation in both chicken lines (Figure 8a). The expression of AQP12 gene was significantly higher in HS compared to TN conditions (*P* < 0.0001), and lower in HWE compared to LWE birds (*P* = 0.048, Figure 8a–d).

### 3.3 Effect of HS on the expression of hypothalamic AVP system in HWE and LWE lines

**FIGURE 9:**
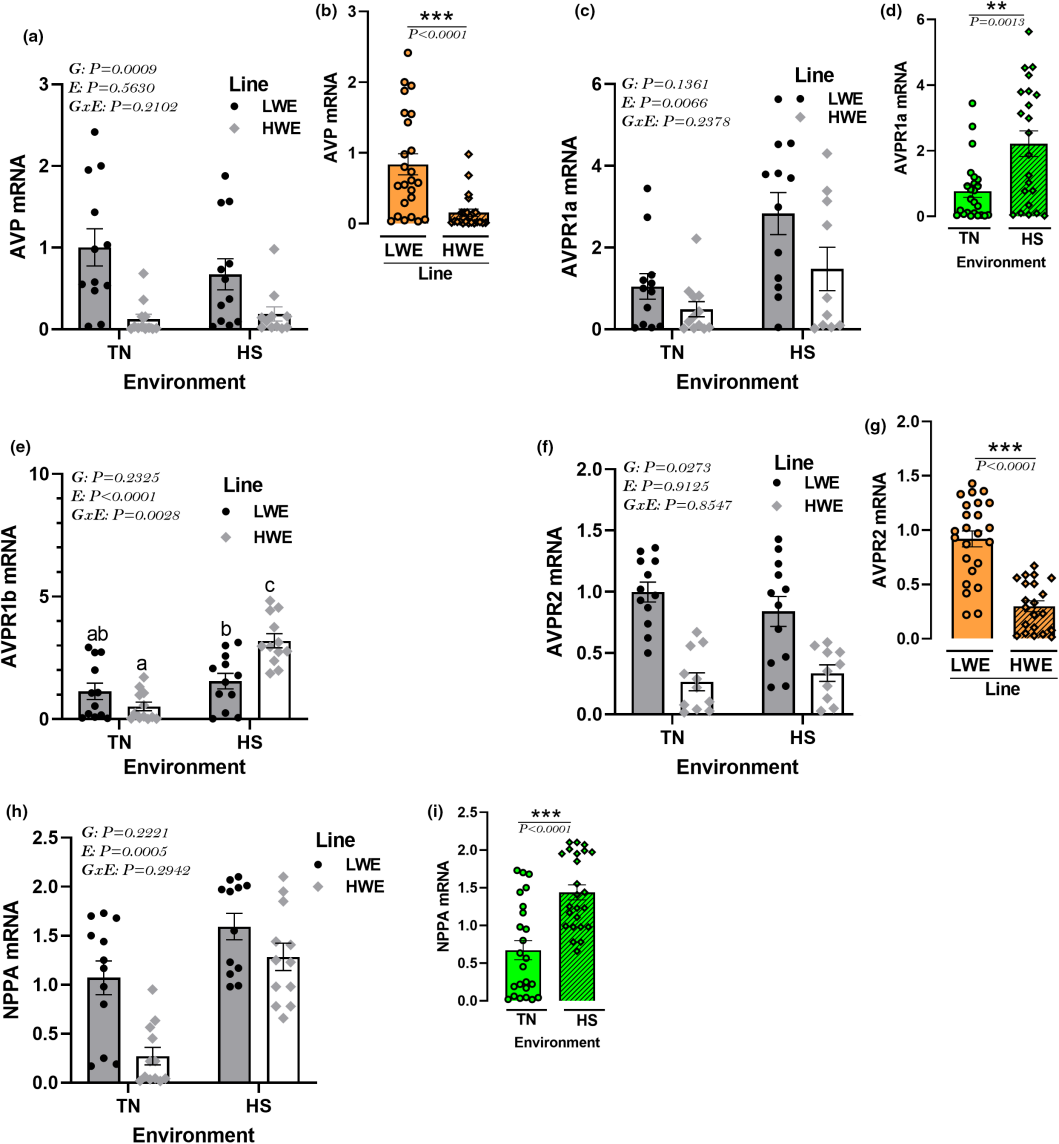
Effects of HS exposure on hypothalamic expression of AVP system in HWE and LWE chickens. Relative expression of AVP1 (a, b), AVPR1a (c, d), AVPR1b (e), AVP2R (f, g), and NPPA (h, i) gene was determined by qPCR and analyzed by 2^−ΔΔCt^ method (Schmittgen & Livak, 2008) using LWE‐TN group as a calibrator. Data are presented as mean ± SD (*n* = 12 birds/group) and analyzed by two‐way ANOVA and Tukey's HSD multiple comparison test. If the G by E interaction is not significant, the main effect (G or E) was analyzed separately by Student's *t*‐test. Asterisk (*) and different letters indicate significant difference at *p* < 0.05. AVP, arginine vasopressin; AVPR, AVP receptor; E, environment; G, genotype or line; GxE, genotype by environment interaction; HS, heat stress; HWE, high‐water efficient; LWE, low‐water efficient; NPPA, natriuretic peptide A; TN, thermoneutral.

The hypothalamic expression of AVP gene and its related receptor AVP2R was significantly downregulated in HWE compared to LWE birds under both environmental conditions (Figure 9a,b,f,g). The expression of its receptor AVPR1a isoform was significantly induced by HS mainly in LWE birds; however, the expression of AVPR1b isoform was upregulated by HS particularly in HWE birds (Figure 9c–e). The hypothalamic expression of NPPA gene was significantly upregulated by HS exposure mainly in HWE birds (Figure 9h,i).

### 3.4 Effects of HS on hypothalamic RAAS expression profile in HWE and LWE lines

**FIGURE 10:**
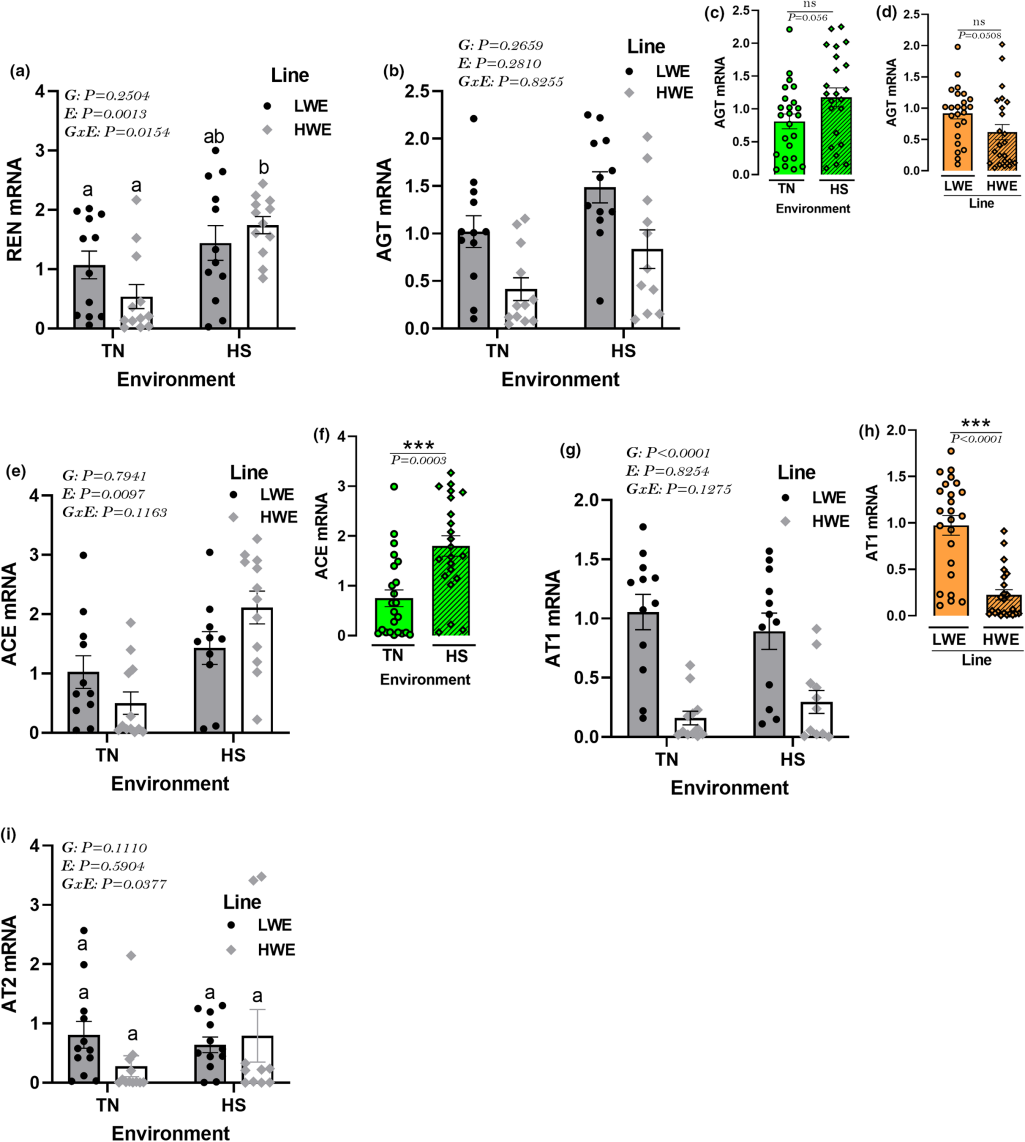
Effects of HS exposure on hypothalamic expression of RAAS system in HWE and LWE chickens. Relative expression of REN (a), AGT (b–d), ACE (e, f), AT1 (g, h), and AT2 (i) gene was determined by qPCR and analyzed by 2^−ΔΔCt^ method (Schmittgen & Livak, 2008) using LWE‐TN group as a calibrator. Data are presented as mean ± SD (*n* = 12 birds/group) and analyzed by two‐way ANOVA and Tukey's HSD multiple comparison test. If the G by E interaction is not significant, the main effect (G or E) was analyzed separately by Student's *t*‐test. Asterisk (*) and different letters indicate significant difference at *p* < 0.05. ACE, angiotensin I‐converting enzyme; AGT, angiotensinogen; AT, angiotensin II receptor; E, environment; G, genotype or line; GxE, genotype by environment interaction; HS, heat stress; HWE, high‐water efficient; LWE, low‐water efficient; TN, thermoneutral.

HS stress exposure significantly upregulated the hypothalamic expression of REN only in HWE birds (Figure 10a). The hypothalamic expression of AGT and ACE was upregulated by HS compared to TN condition (*P* < 0.0001, Figure 10b,c,e,f). The hypothalamic expression of AGT and AT1 was significantly lower in HWE compared to LWE chickens (*P* < 0.0001, Figure 10d,h). The hypothalamic expression of AT2 did not differ between all studied groups (Figure 10i).

### 3.5 Effect of HS on the expression of hypothalamic secretin and calbindin in HWE and LWE lines

**FIGURE 11:**
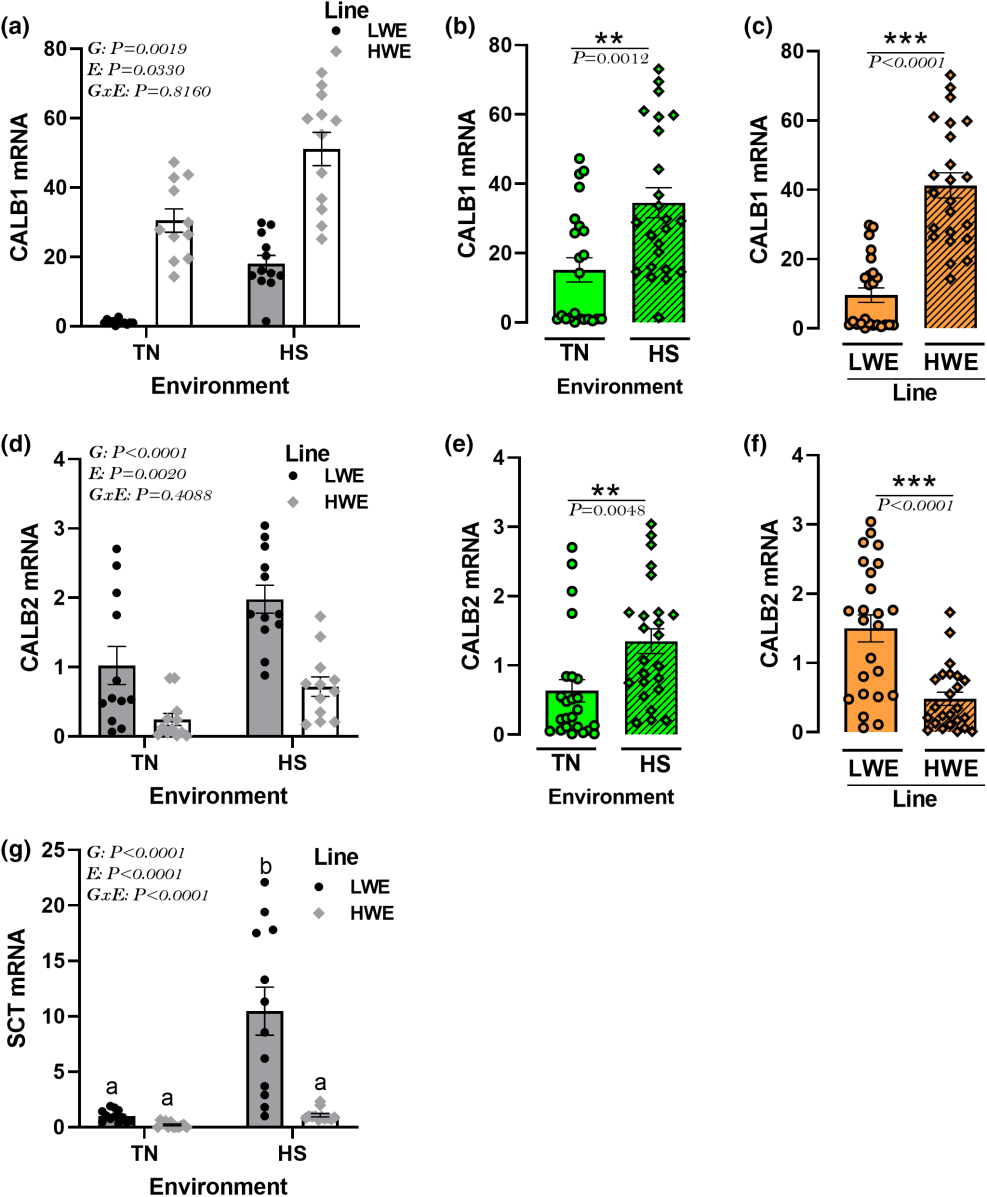
Effects of HS exposure on hypothalamic expression of CALB and SCT in HWE and LWE chickens. Relative expression of CALB1 (a‐c), CALB2 (d‐f), and SCT (g) gene was determined by qPCR and analyzed by 2^−ΔΔCt^ method (Schmittgen & Livak, 2008) using LWE‐TN group as a calibrator. Data are presented as mean ± SD (*n* = 12 birds/group) and analyzed by two‐way ANOVA and Tukey's HSD multiple comparison test. If the G by E interaction is not significant, the main effect (G or E) was analyzed separately by Student's *t*‐test. Asterisk (*) and different letters indicate significant difference at *p* < 0.05. CALB, calbindin; E, environment; G, genotype or line; GxE, genotype by environment interaction; HS, heat stress; HWE, high‐water efficient; LWE, low‐water efficient; SCT, secretin; TN, thermoneutral.

There was no significant environment by genotype interaction for the hypothalamic expression of CALB1 and CALB2 gene. HS significantly upregulated both genes; however, CALB1 expression was significantly higher and CALB2 was significantly lower in HWE compared to LWE birds (Figure 11a–e). The hypothalamic expression of SCT was significantly upregulated by HS only in LWE bids (Figure 11g).

### 3.6 Effect of HS on the hypothalamic expression of TRPVs in HWE and LWE lines

**FIGURE 12:**
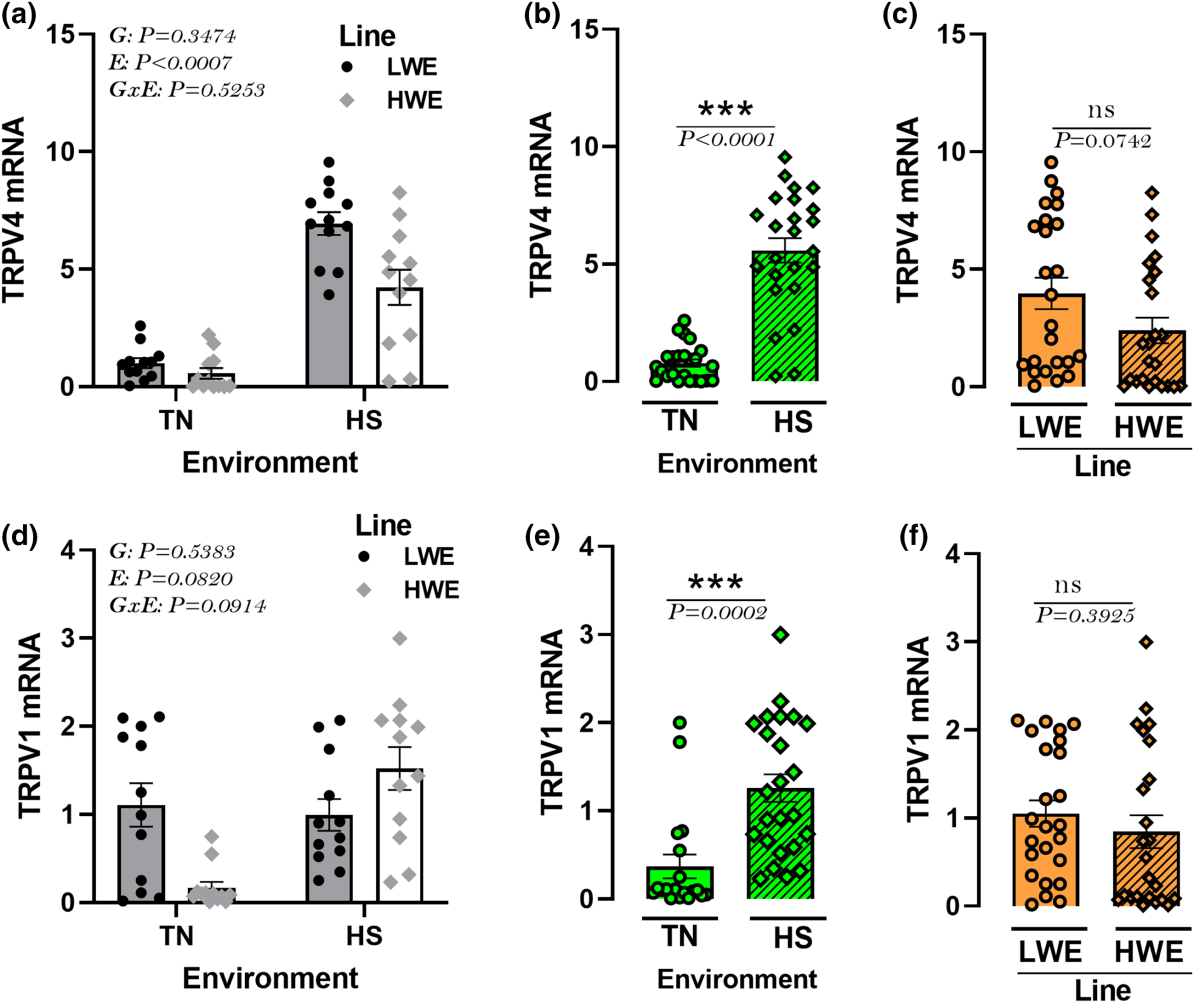
Effects of HS exposure on hypothalamic expression of TRPV family in HWE and LWE chickens. Relative expression of TRPV4 (a–c), and TRPV1 (d–f) gene was determined by qPCR and analyzed by 2^ΔΔCt^ method (Schmittgen & Livak, 2008) using LWE‐TN group as a calibrator. Data are presented as mean ± SD (*n* = 12 birds/group) and analyzed by two‐way ANOVA and Tukey's HSD multiple comparison test. If the G by E interaction is not significant, the main effect (G or E) was analyzed separately by Student's *t*‐test. Asterisk (*) and different letters indicate significant difference at *p* < 0.05. E, environment; G, genotype or line; GxE, genotype by environment interaction; HS, heat stress; HWE, high‐water efficient; LWE, low‐water efficient; SCT, secretin; TN, thermoneutral; TRPV, transient receptor potential cation channel subfamily V.

There was no significant environment by genotype interaction for the hypothalamic expression of TRPV genes. (Figure 12a,d). The hypothalamic expression of TRPV1 and TRPV4 was significantly upregulated by HS compared to TN condition (Figure 12b,e). The expression of both genes remained unchanged between LWE and HWE birds (Figure 12c,f).

## 4 DISCUSSION

In livestock and poultry, although it is not included in the diet formulation, water is the most important nutrient, that is generally required in greater quantity than any other orally ingested substance, and it is consumed mostly as drinking water. A bird, with an average body water content of ~60%–85% (depending on the age, strain, etc.) (Velu et al., 1972), drinks about twice the weight of feed intake, and this ratio can easily quadruple during extreme heat waves (Brake, Chamblee, Schultz, Peebles, & Thaxton, 1992). It is also known that a bird can survive for week(s) without feed, but can live through only a few hours to a few days without water (Bierer et al., 1966), which confirms the critical survival role of water. Therefore, water conservation, saving, and efficiency must be weighed as a priority and considered when discussing poultry production sustainability, especially under current and future planetary boundaries, global warming, and world's emerging water shortage problems (Alcamo, Florke, & Marker, 2007; Xu et al., 2018; He et al., 2021; Tollefson, 2022).

Water efficiency is a vital economic and agricultural trait (Pires et al., 2022), but it is under‐researched and poorly investigated (Rush, 2013) compared to feed efficiency phenotype that as been evolved for decades (Siegel, 2014). Although there are no heritability estimates in the scientific literature for water intake (WI) in poultry and livestock, Bachmanov et al. (2002) and Ramirez and Fuller (1976) reported in mice a WI heritability of 0.65 and 0.44, respectively. Recently, two broiler chicken lines were divergently selected for four generations (F4) in Arkansas for HWE or LWE (Hilts, 2021). Hence, it is important to understand the relationship between the WE and other economically important traits (FI, BWG, FCR, etc.) and define the molecular mechanisms involved in water homeostasis regulation in these lines under thermoneutral and high environmental temperatures, which was the aim of the present study.

As expected, HWE line drank less water compared to their LWE counterparts under both environmental conditions, with curiously no effect of HS where WI of heat‐stressed HWE was lower even than that of TN‐LWE birds. Of particular interest, heat‐stressed HWE line had higher core body temperature and lower feed intake, but a similar BWG as their heat‐stressed LWE counterparts, which resulted in better FCR, WCR, and water (W)/feed (F) ratio. The negative correlation between core body temperature and FI observed in our experimental conditions was not surprising as heat‐stressed birds attempt to limit diet‐induced thermogenesis and reduce endogenous heat production associated with the digestion, absorption, and metabolisms of nutrients (Greene, Cauble, et al., 2021; Lara & Rostagno, 2013). Similarly, the association between WI and FI observed here was not unanticipated because previous studies have reported a high positive phenotypic correlation in rodent and farm animals (Ahlberg et al., 2019; Bachmanov et al., 2002). However, the maintenance of BWG despite the depression of FI and high core body temperature, on the contrary, was intriguing. Although further analyses and investigations are required, it is possible that HWE birds were more efficient in water and feed use (digestion, metabolism, body composition, and thermoregulation) and consequently they were more adapted to hostile environments, without greatly compromising their performance. Moreover, drinking water has been reported to stimulate thermogenesis in humans (Boschmann et al., 2007), and this suggests, although different species, that HWE line had lower energy expenditure, which merits further supportive data. It is also conceivable that HWE chickens maintained efficiently their body water balance by improving metabolic water and reducing water loss, which is favorably and carefully used for various physiological process, such as oxidative phosphorylation, peptide bonds, and enzyme activities (Murphy, 1992), all of which are necessary for growth (Auer et al., 2015; Salin et al., 2015).

Water efficiency is tightly controlled at both central and peripheral levels through complex molecular mechanisms regulating water intake, water retention and excretion, and intermediary metabolism related to water utilization and partitioning (Pollock et al., 2014). Here, as a first step, we sought to determine the expression profile of several systems involved in water homeostasis in the hypothalamus, where the thirst center resides.

The aquaporin (AQP) system, a family of 13 transmembrane proteins, originally discovered as channels and facilitators that regulate water flows between extracellular and intracellular spaces and play a key role in controlling cell volume (Gravelle et al., 2013). They are expressed in different organs/tissues/cells and are classified in three families, including (1) orthodox AQPs (AQP0, 1, 2, 4, 5, 6, and 8) which enable permeabilization of water and some solutes, (2) aquaglyceroporins (AQP3, 7, 9, and 10) which enable permeabilization of water, glycerol, and urea, and (3) super or unorthodox aquaporins (AQP11 and 12) (Huang et al., 2022; Ishibashi et al., 2014). Here, several AQPs were expressed in the hypothalamus and were regulated by HS in a line‐dependent manner. For instance, the expression of hypothalamic AQPs (1, 2, and 3) was upregulated by HS exposure only in LWE but not in HWE line. The gene expression of AQPs (5, 7, 10, 11, and 12) was increased by HS in the hypothalamus of both lines; however, AQP4 expression (mRNA and protein) was downregulated in HS‐LWE birds and AQP9 was not affected by HS. These differential expressions and regulations are probably associated with the selective permeability and function of each AQP. Although the direct effect of these central AQPs on water intake solicits further mechanistic and functional studies, several mammalian AQPs were found in the brain and were suggested to play roles in water movement and cerebrospinal fluid (CSF) formation (Tait et al., 2008). Importantly, some of these AQPs were expressed in choroid plexus, astrocytes, and/or tanycytes, all of which are versatile hypothalamic integrators of energy and fluid metabolism (Badaut & Regli, 2004; Nielsen et al., 1997; Oshio et al., 2005; Rash et al., 1998). Tanycytes, in particular, line the third ventricle and are involved in several key hypothalamic functions including signal transduction, central neuronal activation, and neurohormone secretion (Clasadonte & Prevot, 2018; Goodman & Hajihosseini, 2015; Langlet, 2014), which suggests a complex interdependency between hunger/thirst and water/feed intake.

The AVP or antidiuretic hormone (ADH) is a nonapeptide synthesized by posterior pituitary‐projecting magnocellular AVP neurons of the paraventricular (PVH) and supraoptic nuclei (SON) of the hypothalamus. Upon stimulation, by thirst, dehydration, or elevated systemic osmolality, AVP is released from the axon terminals in the posterior pituitary, enters the circulation (Baylis & Thompson, 1988), and binds to its receptors in target tissues (Bichet, 1996) where it regulates water homeostasis (reabsorption, balance, etc.). At the central levels, the activity of AVP neurons is regulated by feedback mechanism via the lamina terminalis that senses changes in systemic water balance by using AQP receptors (Niermann et al., 2001) and sends (de)polarization downstream that releases AVP. As water leaves the cell, TRPVs sense the mechanical stretch and allow cation influx (Prager‐Khoutorsky et al., 2014), which leads to stimulation of thirst and AVP release. AVP activates the renin‐angiotensin‐aldosterone system (RAAS) and increases angiotensin II secretion, which in turn stimulates water intake (McKinley & Johnson, 2004). The data of the present study suggested that the low water intake in the HWE line was probably mediated through the downregulation of the hypothalamic expression of AVP and its receptor AVPR2, but not AVPR1 isoforms that did not differ between lines. However, the upregulation of the hypothalamic AVPR1a isoform by HS suggested that this receptor is more involved in stress response, which is in accordance with previous studies in rodents (Bielsky et al., 2004) and chickens (Kuenzel et al., 2013). Similarly, the upregulation of the hypothalamic NPPA by HS in our experimental conditions indicated that this gene is more associated with stress responses (Bhattacharya et al., 1996; Wiedemann et al., 2000) rather than fluid and electrolyte homeostasis (Antunes‐Rodrigues et al., 1985).

The understanding of RAAS has expanded tremendously with the discoveries of newer components such as ACE2 (Donoghue et al., 2000) and Massey (MAS) (Santos et al., 2003) genes. The present study was limited to the classical renin‐angiotensin pathway, which has been shown to be ubiquitously expressed and plays a myriad of physiological roles, including water homeostasis regulation (Coble et al., 2014). Basically, angiotensinogen (AGT) is cleaved by renin (REN) to produce angiotensin I (Ang I), which is then cleaved by ACE to produce Ang II, which in turn acts through binding to its receptors, AT1 and AT2. The effect of Ang II on drinking behavior is well known and still serves as a powerful critical control point for accurate forebrain ICV cannula placement for fluid intake studies. Previous mammalian investigations have shown that increased expression of REN, AGT, or Ang II in the brain induced water intake (Coble et al., 2014; Morimoto et al., 2002; Sakai et al., 2007). Here, the classical RAAS system is expressed in the hypothalamus, and the central expression of REN was induced by HS in HWE but not in LWE line. This counterintuitive result suggests that avian central REN, as for many other neuropeptides (Tachibana & Tsutsui, 2016), might have opposite effects on water intake compared to mammals. Furthermore, the two limitations of this study made the REN result nonconclusive. First, the use of the whole hypothalamus might mask the expression of REN in specific nuclei, such as SFO, OVLT, SON, and MnPO that are involved in thirst and water intake. Second, only gene expression has been determined, and protein levels, which are the cellular workhorses, might be different. Interestingly, the lower expression of AGT and AT1 receptor in HWE compared to LWE birds under both environmental conditions, might explain the divergent water intake between the two lines. In support of this hypothesis, specific brain overexpression of AT1 enhanced water intake in rodents (Lazartigues et al., 2008); however, pretreatment with selective antagonists or antisense oligonucleotides abolished water intake (Culman et al., 1999; Sakai et al., 1995).

Although the CALB and SCT systems were originally recognized as intestinal derived chemical factors, their central roles in stress and water homeostasis have recently attracted research attention, yet their understanding is far to be complete. CALBs are calcium‐binding, sensor‐, and transport‐proteins, primarily characterized in the gut (Desplan et al., 1983). Several studies showed that CALBs are also expressed in brain vasopressin neurons (Arai et al., 1999), affected by stress (Nowak et al., 2010), and interact with AT1 receptor (Yu et al., 2023). Secretin, a member of the secretin‐glucagon family, was originally found to be secreted by the duodenal S cells in the crypts of Lieberkuhn (Hacki, 1980) and has been reported to exert pleiotropic effects from pH regulation, gastric acid secretion, to water homeostasis (Chu et al., 2009; Chu et al., 2011). Here, as expected, HS increased the hypothalamic expression of CALBs, which is in agreement with previous studies (de Moraes et al., 2021; Ebeid et al., 2012). However, the differential expression of CALBs (upregulation of CALB1 and downregulation of CALB2) in HWE line is puzzling. This suggests that these two CALBs might have different central roles in chickens as previously reported in human entorhinal cortex (Mikkonen et al., 1997). Similarly, the line‐dependent induction of the hypothalamic SCT by HS is also intriguing and merits further in‐depth investigation.

The last system tested in our study was the vanilloid TRP (TRPV) subfamily, which is a nonselective cation channel with high calcium permeability and was initially identified as a receptor for capsaicin, the pungent compound of chili pepper (Caterina et al., 1997). It is involved in pain pathways and is activated by various stimuli such as noxious heat (Caterina et al., 1997), external pH (Dhaka et al., 2009), and mechanical pain (Ross, 2003). In the last decade, several groups have reported a role for the TRPV subfamily in the regulation of water homeostasis (Ciura & Bourque, 2006; Kinsman et al., 2014; Liedtke & Friedman, 2003; Sharif‐Naeini et al., 2008). Here, hypothalamic TRPV4 and TRPV1 expression did not differ between the two lines, but it was induced by HS, indicating a role in central thermoreception and thermal homeostasis (Wechselberger et al., 2006), but not in water intake regulation. It is worth noting that he TRP family is subdivided into seven subfamilies: TRPC (canonical), TRPV (vanilloid), TRPM (melastatin), TRPP (polycystin), TRPML (mucolipin), TRPA (ankyrin), and TRPN (NOMPC‐like); the latter is found only in invertebrates and fish (Nilius & Owsianik, 2011). Each multigene subfamily encodes integral membrane proteins that function as ion channels, some of them could potentially be involved in integrated thermal, osmotic and/or water homeostasis regulation in chickens, which merits further investigations.

In summary, this is the first study reporting growth performances and expression profile of genes associated with water homeostasis in the hypothalamus of two broiler lines divergently selected for HWE or LWE. HWE line exhibited better FCR, WCR, and W/F ratio and maintained a similar BWG despite the low water intake. This is particularly interesting for poultry production sustainability as future water may not be as readily available. In addition, several key genes showed a line‐ or environment‐dependent expression, which open new vistas for future research for subsequent identification of molecular signatures for efficient marker‐assisted selection in poultry breeding for water/feed efficiency and thermo‐resistance.

## AUTHOR CONTRIBUTIONS

Sami Dridi conceived and designed the study. Sami Dridi provided the reagents. Elizabeth S. Greene, Travis Tabler, Kentu Lassiter, Sara Orlowski, Walter G. Bottje, and Sami Dridi conducted the experiments and analyzed the data. Kentu Lassiter performed the statistics. Loujain Aloui performed the qPCR analysis. Sami Dridi wrote the final paper with a critical review by all coauthors.

## FUNDING INFORMATION

This study was supported by a grant from USDA NIFA Sustainable Agriculture Systems (#2019 69012‐29905) to S.D. and W.B. Loujain Aloui was supported by an internship from the same USDA‐SAS grant.

## Data Availability

Raw data generated during the current study are available on reasonable requests from the corresponding author.
